# Perceptions of insulin use in type 2 diabetes in primary care: a thematic synthesis

**DOI:** 10.1186/s12875-018-0753-2

**Published:** 2018-05-22

**Authors:** Kathy Ellis, Henrietta Mulnier, Angus Forbes

**Affiliations:** Florence Nightingale Faculty of Nursing, Midwifery and Palliative Care, King’s College London, 57 Waterloo Road, London, SE1 8WA UK

**Keywords:** Insulin, Type 2 diabetes, Patients, General practice, Primary care, Perceptions

## Abstract

**Background:**

Increasing numbers of patients with type 2 diabetes mellitus are progressing to insulin therapy, and despite its potency many such individuals still have suboptimal glycaemic control. Insulin initiation and intensification is now often conducted by Practice Nurses and General Practitioners in many parts of the UK. Therefore, gaining insight into perspectives of patients and primary care clinicians is important in determining self-management and engagement with insulin. A thematic synthesis of studies was conducted exploring the views and experiences of people with type 2 diabetes and of healthcare professionals on insulin use and management in the context of primary care.

**Methods:**

Protocol based systematic searches of electronic databases (CINAHL, Cochrane Library, EMBASE, MEDLINE, PsycINFO, and Web of Science) were performed on 1 October 2014 and updated on 31 March 2015, to identify studies that identified the views and experiences of adults with type 2 diabetes or primary care clinicians on the use of insulin in the management of type 2 diabetes. Studies meeting the review inclusion criteria were critically appraised using the CASP qualitative research checklist or Barley’s checklist for survey designs. A thematic synthesis was then conducted of the collected studies.

**Results:**

Thirty-four studies were selected. Of these, 12 used qualitative interviews (nine with patients and three with healthcare professionals) and 22 were survey based (14 with patients, three with healthcare professionals, and five with both). Twelve key themes were identified and formed three domains, patient perceptions, healthcare professional perceptions, and health professional-patient relationships. The patient-centred themes were: insulin-related beliefs, social influences, psychological factors, hypoglycaemia, and therapy barriers. The clinician-related themes were: insulin skills of general practitioners, healthcare integration, healthcare professional-perceived barriers, hypoglycaemia, and explanations for adherence. Healthcare professional-patient relationship themes were drawn from the perspectives of patients and from clinicians.

**Conclusions:**

This review reveals multiple barriers to optimal insulin use in primary care at both the patient and healthcare professional levels. These barriers indicate the need for multimodal interventions to: improve the knowledge and competencies of primary care professionals in insulin use; provide more effective patient education and self-management support; and introduce integrated insulin support systems.

**Electronic supplementary material:**

The online version of this article (10.1186/s12875-018-0753-2) contains supplementary material, which is available to authorized users.

## Background

Many patients living with type 2 diabetes mellitus (T2DM) require insulin as an adjunct to lifestyle interventions and oral hypoglycaemic agents [1–3]. As the population of people affected by T2DM increases, the number of those requiring insulin therapy also increases. While insulin therapy was traditionally managed by specialist diabetes services it is now largely managed in primary care by Practice Nurses (PNs) and General Practitioners (GPs) [4–7].

While insulin is a very effective glucose lowering therapy, it has been shown that many people with insulin treated T2DM have poor glycaemic control (8, 9, 10). There are a number of factors that may explain this problem. Firstly, that there may be some clinical inertia in introducing insulin, as it is often introduced after patients have had poor glycaemic control for some time. Secondly, it has been suggested that both patients and clinicians are reluctant to start insulin due to what has been termed psychological insulin resistance [4, 8, 9]. There is a perception that insulin: represents the last line of treatment and is associated with failure; increases the patient’s self-management burden; and imposes hazards such as hypoglycaemia and weight gain [10]. Hence, despite improvements in insulin delivery and support systems, insulin is often not used optimally in primary care settings, increasing the patients risks of complications. [1, 11, 12]. Therefore, developing a better understanding as to what factors influence insulin use in primary care is important to shape interventions to enhance insulin management in this setting.

While previous reviews have explored some of the factors related to why insulin use often fails to deliver good outcomes [10, 13–18], these factors have not been considered systematically in the context of the management of T2DM in primary care from both the patient and healthcare professional perspectives collectively. In this paper, we present a synthesis of the views of patients already established on insulin treatment, and health professionals within primary care, to elicit mechanisms that may explain the use of the therapy and to consider how these may be addressed through more optimal strategies for insulin management in this population.

The aim of the review was to identify and synthesise studies exploring the views and experiences of people with insulin treated T2DM and healthcare professionals (HCPs) within the context of primary care on insulin use to elicit the factors that contribute to sub-optimal insulin use in primary care. The review addressed the following questions:

1. What are the perceptions and experiences of people with T2DM in relation to insulin treatment and use?
2. What are the perceptions and experiences of primary care HCPs on insulin treatment use in people with T2DM?
3. What potential patient-professional interactions impact on insulin use in T2DM?

## Methods

Thematic synthesis is a process of identifying new insights by integrating data from original studies and is one of a range of methods available for synthesizing diverse forms of evidence [13, 19–26]. Thematic synthesis generally refers to the integration of findings from qualitative studies, but it has also been used to integrate quantitative and qualitative research; including studies using descriptive or interpretive phenomenological approaches [20, 24, 27–29]. While integrating findings derived from different methods can be challenging and subject to criticism [20, 23, 26, 30], the approach can allow a more expansive interpretation of what is known about the studied phenomena. In this review a narrative synthesis was used to identify the key themes, as an established method for integrating across study types [20, 21, 23–25, 27–29]. Thomas & Harden’s [26] approach was observed as a framework for the analysis, but with the inclusion of quantitative in addition to qualitative studies. This progressed in three steps.

### Step 1. Identification of studies

Reports of qualitative and quantitative studies addressing the review questions were identified from peer-reviewed journals, conference reports and theses.

#### Inclusion criteria

Papers were required to report on studies of insulin-related experiences and/or perceptions in either adults aged ≥18 years with insulin treated T2DM or of primary care HCPs. It was also required that the study should focus on patients already receiving insulin therapy, and not on insulin initiation. Study design eligibility included qualitative and descriptive quantitative studies such as surveys and included those with lower or unreported response rates (which may not be apparent especially in web-based surveys).

#### Search strategy

A protocol-based search was performed by KE on 1 October 2014 and updated on 31 March 2015, to retrieve articles from electronic databases including CINAHL, Cochrane Library, EMBASE, MEDLINE, PsycINFO and Web of Science. The search was structured by terms for T2DM; insulin therapy; and primary care. Discrete searches were also performed with terms for primary care HCPs. The electronic database search strategy can be viewed in Additional file 1. There was no limit to the year of publication but they were required to be published in English. The search was supplemented with: open web-based searches (Google Scholar and EthOs); citation and key author searching; and hand searches of journals. Using EndNote X7 bibliography software, titles and abstracts were screened for inclusion. Full-text articles of the remaining reports were then fully assessed for eligibility by KE before the final selection. To help ensure lack of bias, AF and HM reviewed the search strategy, studies generated, and the final selection; and agreement was reached between the reviewers. In the absence of a standard guideline for reporting thematic syntheses with combined qualitative and quantitative studies [27], the principles of ENTREQ and PRISMA were applied and their study reporting checklists used [31, 32].

#### Step 2. Content extraction and appraisal

Data were extracted by KE using standardized extraction tools [33, 34], one for qualitative designs and one for surveys. Methodological quality and risk of bias for studies with qualitative designs were assessed using the Critical Appraisal Skills Programme (CASP) qualitative research checklist [35] whilst the survey studies were assessed with Barley et al.’s tool [28]. A score of one was given where the study answered most parts of the appraisal tools’ questions.

#### Step 3. Synthesis of the extracted content

The included studies were subjected to a thematic synthesis by KE, and reviewed by HM and AF until agreement was reached. This was undertaken in 3 stages.

##### Stage 1

Findings of the qualitative studies were scrutinized for concepts, themes and authors’ interpretations relating to managing insulin treated T2DM. Themes were developed inductively, and the text was then coded manually. Next, the main themes from the quantitative studies were identified, and categorized separately.

##### Stage 2

Descriptive themes and sub-themes from the qualitative studies were inductively developed from the coded text and organized into two primary thematic frameworks one for patients and the other for HCPs. The main finding clusters (including thematic analysis of survey comments) from the quantitative studies were then mapped onto these frameworks, to integrate themes from the different data sources.

##### Stage 3

Analytical themes were then generated from the descriptive themes for patients and HCPs, to further address the aims of the review, and to identify areas for further research.

## Results

### Summary of the selected studies

The search strategy identified 147 papers for screening, 70 of which were fully appraised for eligibility. Though the numbers retrieved were lower than anticipated, this was attributed to the inclusion criteria with its focus on experiences and perceptions of T2DM participants already established on insulin and primary care HCPs. Thirty-four of the screened studies fulfilled the inclusion criteria with 36 being rejected with reasons (see Table 1.). Of the included studies, 12 were qualitative [36–47] (nine with patient participants and three with HCPs) and 22 were surveys [48–69] (14 with patient participants, three with HCPs and five with both patients and HCPs). Five of the surveys were discrete reports from two larger studies.

**Table 1:** Rejected Studies with Reasons

Author & Reference	Year	Reason for Rejection
Aloumanis [83]	2013	The focus is on clinical outcomes rather than perceptions and experiences.
Bahrmann [9]	2014	The focus is on psychological insulin resistance in insulin naïve patients compared to those established on insulin.
Balkau [84]	2012	The patient participants are insulin naïve.
Beresford [85]	2011	Insufficient data specific to insulin treated T2DM.
Beverly [86]	2012	Insufficient data specific to insulin treated T2DM.
Brod [87]	2013b	It was not possible to differentiate data specific to insulin treated T2DM.
Carbone [88]	2007	It was not possible to differentiate data specific to insulin treated T2DM.
Chai [89]	2012	Conference abstract only. No other data available.
Chai [90]	2013	Conference poster only. No other data available.
Chai [91]	2014	Conference abstract only. No other data available.
Chan [92]	2014	The patient participants are insulin naïve.
Choudhury [93]	2014	It was not possible to differentiate data specific to insulin treated T2DM.
Cramer & Pugh [94]	2005	The focus is on insulin prescriptions issued and not on perceptions or experiences.
Gaborit [95]	2011	The focus is on knowledge rather than experiences of insulin adjustment.
Hermanns [96]	2010	The focus is on comparing barriers of insulin naïve patients.
Hinder & Greenhalgh [97]	2012	Insufficient data specific to insulin treated T2DM.
Frei [98]	2012	The focus is on clinical characteristics and demographics.
Hunt [99]	1998	Insufficient data specific to insulin treated T2DM.
Khattab [100]	2010	The focus is on clinical characteristics and demographics.
Lai [101]	2007	It was not possible to differentiate data specific to insulin treated T2DM.
Lakkis [102]	2013	The focus is on attitudes of clinicians towards initiating insulin.
Mollem [103]	1996	It was not possible to differentiate data specific to insulin treated T2DM.
Morris [104]	2005	Patients only recently initiated with insulin therapy.
Munro [73]	2013	There is no information specific to insulin treated T2DM.
Oliveria [105]	2007	The focus is on patients who did not start or continue insulin therapy.
Peyrot [106]	2005	Patient participants are insulin naïve. Perceptions of clinicians relate to insulin initiation.
Peyrot [107]	2006	Insufficient data specific to insulin treated T2DM.
Peyrot [108]	2013	Insufficient data specific to insulin treated T2DM.
Pooley [109]	2001	No data specific to insulin treated T2DM.
Ritholz [72]	2011	Insufficient data specific to insulin treated T2DM
Shiu & Wong [110]	2000	It was not possible to differentiate data specific to insulin treated T2DM.
Thomson [111]	1991	The focus is on knowledge rather than experiences or perceptions of hypoglycaemia.
Wendel [112]	2014	The focus is on incidence of hypoglycaemia and prescribing behaviour rather than perceptions of hypoglycaemia
Wong [113]	2011	Patients were insulin naïve.
Yoshioka [74]	2014	The focus is on insulin initiation.
Zafar [8]	2015	Insufficient data specific to insulin treated T2DM.

The qualitative studies included data from 173 patients with insulin treated T2DM, aged 23–90 years. The HCP studies included: GPs (*n* = 65); endocrinologists (*n* = 2), PNs (*n* = 8), diabetes nurse educators (*n* = 3) and pharmacist (*n* = 1). Their methodologies varied and included focus groups; and in-depth and semi-structured interviews conducted mainly face-to-face, with one study using both telephone interviews and focus groups. The majority of these studies used a thematic, descriptive approach, with some using other methods such as grounded theory, theoretical frameworks, and interpretative phenomenological methods of inquiry.

The quantitative studies were survey-based with mainly cross-sectional designs and included: 13,476 patients with T2DM receiving insulin, aged 41–99 years; GPs (*n* = 4,176); diabetes consultants (2,192); general physicians (*n* = 166); general nurses (*n* = 51); Diabetes Specialist Nurses (DSNs) (*n* = 50); and diabetes educators (*n* = 100). The majority of the surveys were web-based with some being undertaken as face-to-face questionnaires or by telephone.

A number of studies took place in multiple sites and in two or more countries. The qualitative research sites included: Asia (*n* = 4), Australia (*n* = 1), Europe (*n* = 7), New Zealand (*n* = 1), and North America (n = 1). Those of the quantitative studies included: Asia (*n* = 7), Australia (*n* = 1), Europe (*n* = 15), North America (*n* = 12), South America (*n* = 2), and South Africa (*n* = 1).

**Fig. 1:**
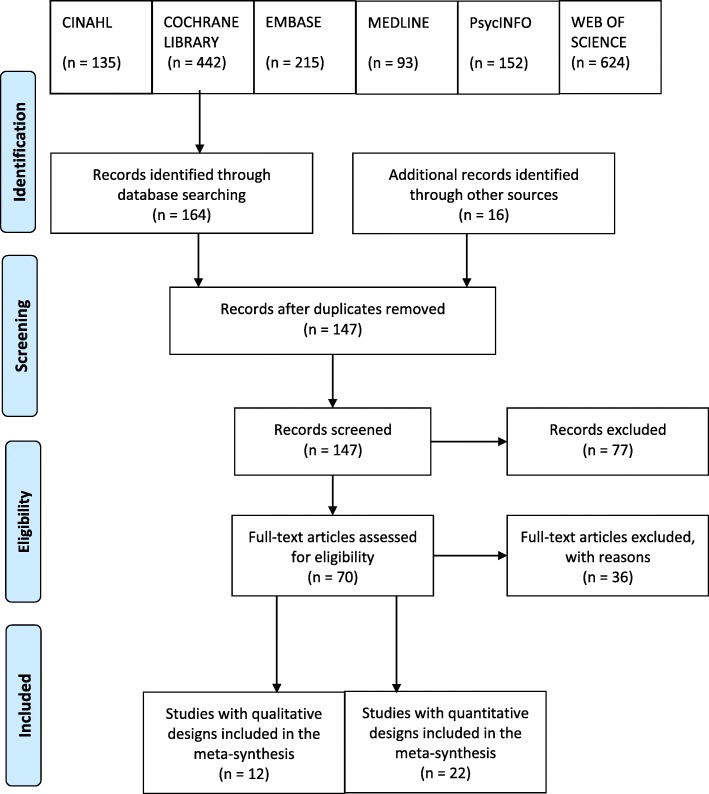
PRISMA Flow Diagram Illustrating the Selection Process

The methodology and reporting quality of the qualitative studies was generally good with scores ranging from 8 to 10; the quantitative studies were of moderate strength with scores ranging from 3 to 7. Tables 2, 3, 4. present an overview of the included studies, and the selection process is shown in a PRISMA flow chart in Fig. 1. The appraisal scores are presented in Additional file 2. Where available, the survey response-rate has been entered although this was not available in many of the surveys which were reported online. Survey limitations include pharmaceutical company support, recruitment bias with sampling from research panels and self-selection in online surveys; and self-reporting of clinical data. However, it was decided to include the surveys because of their contribution to the overall themes of the synthesis.

**Table 2:** Overview of the Included Qualitative Studies with Patient Participants

Author & Reference	Year	Country	Diabetes Type	Aim	Sample and Setting	Data Collection	Data Analysis
Abu Hassan [36]	2013	Malaysia	Insulin T2DM	To explore patients’ reasons for accepting insulin and their initial barriers.	Patients with insulin T2DM (*n* = 21) Primary Care Clinic	In-depth interviews Focus groups	Thematic analysis
Brod [37]	2014	Canada, China & Germany	T1DM & Insulin T2DM	To examine unintentional insulin dosing and injection irregularities due to forgetting among people with diabetes.	Patients with T1DM (*n* = 22) Insulin T2DM (*n* = 42) At least twice in the last three months of forgetting injection, or time/amount taken, or questioning if insulin was taken. Research recruitment databases	Telephone interviews Focus groups	Thematic analysis with grounded theory
Brown [38]	2007	UK	Insulin T2DM & Non-Insulin T2DM	To gain an understanding of how health beliefs influence how African-Caribbeans manage their T2DM.	T2DM adults (n = 16) Insulin T2DM (n = 6) Self-help groups and GP practices Inner-city African-Caribbean community	In-depth interviews	Thematic analysis
Browne [39]	2013	Australia	Insulin T2DM & Non-Insulin T2DM	To explore the social experiences of adults with T2DM, focusing on the perception & experience of diabetes- related stigma.	T2DM adults (*n* = 25) Insulin T2DM (n = 5) State diabetes organisation	Semi-structured interviews	Inductive thematic analysis
Hortensius [40]	2012	Netherlands	T1DM & Insulin T2DM	To investigate patients’ perspectives of SMBG & all relevant aspects influencing SMBG.	Insulin treated DM patients (*n* = 28) T1DM (*n* = 13) T2DM (*n* = 15) Outpatient clinic (T1DM) GP practices (T2DM)	In-depth interviews	Thematic analysis with grounded theory.
Janes [41]	2013	New Zealand	Insulin T2DM	To better understand barriers to glycaemic control from the patient’s perspective.	Insulin treated patients T2DM (n = 15) Diabetes clinic	Semi-structured interviews.	Thematic analysis with a patient-centred framework. Interpretative phenomenological method of inquiry
Jenkins [42]	2011	UK and Ireland	Insulin T2DM	To explore participants’ experiences of intensifying insulin therapy during the Treating to Target in T2DM (4-T) trial.	T2DM patients (*n* = 41) Whose insulin was intensified in 4-T trial. Primary care	In-depth interviews	Thematic analysis with grounded theory.
Ong [43]	2014	Malaysia	Insulin T2DM	To explore the barriers and facilitators to SMBG, in insulin T2DM patients.	Insulin treated T2DM patients (n = 15) Primary care clinic	Semi-structured interviews	Inductive thematic analysis
Vinter-Repalust [44]	2004	Croatia	Insulin T2DM & Non-insulin T2DM	To explore patients’ attitudes, thoughts, & fears connected with their illness; expectations of the healthcare system; and problems while adhering to the therapeutic regime.	Patients with T2DM (*n* = 49) Insulin T2DM (n = 13) General practice	Focus group discussions.	Inductive thematic analysis

Key: *DSN* diabetes specialist nurse, *PN* practice nurse, *GP* general practitioner, *HCP* health care professional, *OHAs* oral hypoglycaemic agents, *PCPs* primary care physicians, *QOL* quality of life, *SMBG* self-monitoring of blood glucose, *T1DM* type 1 diabetes mellitus, *T2DM* type 2 diabetes mellitus, *Insulin T2DM* insulin treated type 2 diabetes mellitus

**Table 3:** Overview of the Included Qualitative Studies with HCP Participants

Author & Reference	Year	Country	Aim	Sample and Setting	Data Collection	Data Analysis
Goderis [45]	2009	Belgium	To evaluate barriers and facilitators to high quality diabetes care by GPs participating in a quality improvement programme promoting compliance with international guidance.	GPs participating in the programme (n = 20) General Practice.	Semi-structured interviews	Thematic analysis with an implementation and behavioural change model.
Jeavons [46]	2006	UK	To determine doctors’ and nurses’ attitudes and beliefs on treating T2DM with less than ideal control.	GPs (*n* = 15) Practice Nurses (n = 8) General Practice	Focus groups.	Thematic analysis with grounded theory.
Lee [47]	2013	Malaysia	To explore the views of Malaysian healthcare professionals on the barriers faced by patients using insulin.	Primary care doctors (*n* = 20) Family medicine specialists (*n* = 10) Policymakers (n = 5) Diabetes educators (*n* = 3) Endocrinologists (n = 2) Pharmacist (n = 1) Primary & secondary care	In-depth interviews Focus group discussions	Inductive thematic analysis

Key: *DSN* diabetes specialist nurse, *PN* practice nurse, *GP* general practitioner, *HCP* health care professional, *OHAs* oral hypoglycaemic agents, *PCPs* primary care physicians, *QOL* quality of life, *SMBG* self-monitoring of blood glucose, *T1DM* type 1 diabetes mellitus, *T2DM* type 2 diabetes mellitus, *Insulin T2DM* insulin treated type 2 diabetes mellitus

**Table 4:** Overview of the Included Quantitative Studies with Patients, HCPs, or Patients and HCPs

Author & Reference	Year	Country	Diabetes Type Patients or HCPs	Aim	Sample and Recruitment	Data Collection
Ary [48]	1986	USA	Insulin T2DM & Non-insulin T2DM Patients only	To assess levels of regime adherence and reasons for non-adherence in T1DM and T2DM	Patients with T1DM (*n* = 24) Non-insulin T2DM (*n* = 125) Insulin T2DM (*n* = 59) Recruited by doctors, newspaper adverts & American Diabetes Association meetings	Face-to-face Questionnaire
Brod [49]	2012a	USA, Canada, Japan, Germany, UK and Denmark	Insulin T2DM Patients and HCPs	To estimate the prevalence of self-treated hypoglycaemia in patients using basal analogues. To identify demographic treatment-related and behavioural risk factors. To describe patient and physician responses to these in the Global Attitude of Patients and Physicians 2 (GAPP2) study.	T2DM Patients using basal insulin analogues (*n* = 3,042) Physicians (*n* = 1,222): Specialists (45%) PCPs (55%) Online research panel	Cross-sectional online questionnaire
Brod [50].	2012b	USA, Canada, Japan, Germany, UK and Denmark	Insulin T2DM Patients and HCPs	To describe basal insulin analogue dosing irregularities; the effect on patient functioning, well-being and management; and the identification of patients most at risk in the GAPP2 study.	T2DM Patients using basal insulin analogues (n = 3,042) Physicians (n = 1,222): Specialists (45%) PCPs (55%) Online research panel	Cross-sectional online questionnaire
Brod [51]	2012c	USA, UK, Germany and France	T1DM, Insulin T2DM & Non-insulin T2DM Patients only	To determine how non-severe nocturnal hypoglycaemic events (NSNHEs) affect diabetes management, sleep quality, functioning, and to assess if these impacts differ by diabetes type or country.	T1DM and T2DM patients (*n* = 1086) who experienced NSNHE in the last month: T1DM (*n* = 676) Non-Insulin T2DM (*n* = 124) Insulin T2DM (*n* = 286) Online venues	Web-based survey
Brod [52]	2013a	USA, UK, Germany, Canada, France, Italy, Spain, Netherlands and Sweden	T1DM, Insulin T2DM & Non-insulin T2DM Patients only	To explore the burden and impact of NSNHEs on diabetes management, patient monitoring and well-being to better understand the role NSNHEs play in caring for people with diabetes and to facilitate optimal diabetes treatment strategies.	Patients (*n* = 2,108) with: T1DM or T2DM. T1DM (*n* = 692) Non-insulin T2DM (*n* = 543) Insulin T2DM (*n* = 873) Online venues	Web based survey
Cefalu [53]	2008	USA, Mexico, UK, France, Germany, Spain and Brazil	Insulin T2DM & Non-Insulin T2DM Patients only	To understand patients’ perspectives to achieving good glycaemic control and determine how their perceptions of insulin may affect their decisions to initiate or intensify insulin.	T2DM adults (*n* = 1,444) of which: Insulin T2DM (*n* = 469) Online databases	Structured online and telephone survey.
Cuddihy [54]	2011	Germany, Japan, Spain, Turkey, UK and USA	HCPs Only	To investigate the opinions of PCPs and diabetes specialists on their perceived role in tackling T2DM and the challenges they face, particularly to insulin intensification.	Diabetes specialist physicians (*n* = 300) PCPs (*n* = 300) Recruited by telephone and online panels	Online survey
Diago-Cabezudo [55]	2013	Europe	T1DM & Insulin T2DM Patients only	To evaluate the effects of hypoglycaemia on the lives of patients with DM and determine if SMBG to prevent hypoglycaemic is an appealing and widely accepted concept.	Insulin treated patients (*n* = 1,848) T1DM (n = 924) Insulin T2DM (*n* = 924) Online databases	Online survey
Fulcher [56]	2014	Argentina, Australia, Brazil, Israel, Mexico and South Africa	T1DM, Insulin T2DM & Non-insulin T2DM Patients only	To understand the impact of nocturnal and daytime non-severe hypoglycaemic events on healthcare systems, work productivity & QOL in T1DM or T2DM.	T1DM (*n* = 64) Non-insulin T2DM (*n* = 76) Insulin T2DM (*n* = 160) Recruited from online panels and by HCPs	Online and face-to-face surveys
Leiter [57]	2005	Canada	T1DM & Insulin T2DM Patients only	To assess impact of mild, moderate and severe hypoglycaemia and fear of future episodes on patients with T1DM or insulin-treated T2DM	Adults with insulin treated T2DM (*n* = 335) T1DM (*n* = 202) insulin T2DM (*n* = 133) Diabetes Clinics	Self-administered questionnaire
Leiter [58]	2014	Canada	Insulin T2DM Patients and HCPs	To assess the frequency and impact of dosing irregularities and self-treated hypoglycaemia in T2DM patients treated with insulin analogues in the GAPP2 study.	Patients with Insulin treated T2DM (*n* = 156) Physicians (n = 202) Of which: PCPs (*n* = 160) Specialists (*n* = 42) Online panels and HCP registers	Online survey
Mehmet [59]	2015	UK	T1DM & Insulin T2DM Patients only	To determine if patients report problems with injecting insulin/SMBG in front of others and explore reasons why.	Insulin T2DM (*n* = 27) T1DM (*n* = 49) Hospital Clinic	Self-completed questionnaire
Mitchell [60]	2013	UK	Insulin T2DM & Non-insulin T2DM Patients only	To characterize hypoglycaemic events in T2DM and assess the relationship between the experiences and health outcomes.	T2DM adults (*n* = 1,329) of which: Insulin T2DM (*n* = 301) Research survey panel	Longitudinal online survey
Mollema [61]	2001	Netherlands	T1DM & Insulin T2DM Patients only	To examine functioning and self-management of insulin treated patients suffering from extreme fear of self-injecting and/or fear of self-testing.	Patients with insulin treated diabetes (*n* = 1,275) of which: T1DM (*n* = 740) T2DM (*n* = 535) Randomly drawn from the Dutch Diabetes Association	Cross-sectional postal questionnaire
Mosnier-Pudar [62]	2009	France	Insulin T2DM & Non-insulin T2DM Patients only	To describe T2DM from the patient’s standpoint in a representative French panel in 2008.	T2DM Patients (*n* = 1,092) of which: Non-Insulin (*n* = 885) Insulin T2DM (*n* = 207) From a polling institute in France	Postal questionnaire
Peyrot [63]	2012a	China, Japan, USA, Germany, Spain, France, Turkey & UK	T1DM & Insulin T2DM Patients only	To examine factors associated with insulin injection omission/ non-adherence in the Global Attitude of Patients and Physicians (GAPP) Study.	Insulin treated DM adults (*n* = 1,530) of which: T1DM (*n* = 110) T2DM (*n* = 1,420) Research panels	Cross-sectional telephone survey
Peyrot [64]	2012b	China, Japan, USA, Germany, Spain, France, Turkey & UK	T1DM & Insulin T2DM Patients and HCPs	To examine patient and physician beliefs regarding insulin therapy and degree to which patients adhere to insulin regimes in the GAPP Study.	Insulin treated DM adults (*n* = 1,530) of which: T1DM (*n* = 180) T2DM (*n* = 1,350) Physicians (*n* = 1,250) of which Specialists (*n* = 600) PCPs (*n* = 650) Research panels	Cross-sectional telephone survey
Rubin [65]	2009	USA	T1DM & Insulin T2DM Patients and HCPs	To compare patients’ perceptions of injection-related problems with clinicians’ estimates of those problems.	Insulin treated adults (*n* = 501) of which T2DM (*n* = 385) PCPs (*n* = 101) Endocrinologists (n = 100) Diabetes Educators (*n* = 100) Chronic illness panel, Medical Register and Research database.	Online survey
Shiu [66]	2004	Hong Kong	Insulin T2DM Patients only	To examine the relationship between a sense of coherence, fear of hypoglycaemia and metabolic control to identify whether other variables including age, hypoglycaemic experience and adherence to self-care practice, confounded the findings from two Swedish studies.	Insulin treated T2DM adults (*n* = 72) Diabetes Centre	Cross-sectional face-to-face questionnaire
Siminerio [67]	2007	USA	HCPs only	To examine nurse and physician perceptions of nurse involvement in diabetes care.	General Nurses(n = 51) DSNs (n = 50) Generalist Physicians (n = 166) Diabetes Specialist Physicians (*n* = 50) Professional directories and listing	Cross-sectional survey conducted face-to-face or by telephone.
Van Avendonk [68]	2009	Netherlands	HCPs only	To investigate the organisation of insulin therapy in general practice and assess factors associated with providing insulin in T2DM patients.	Dutch GPs (n = 1,621) University Medical Centre database.	Postal questionnaire
Zambanini [69]	1999	UK	T1DM & Insulin T2DM Patients only	To assess: prevalence of phobia and anxiety-related to insulin injections; association between insulin injection anxiety symptoms with level of general anxiety in the study group; and evaluate their influence of, on glycaemic control.	Insulin treated patients (*n* = 115) of which: T1DM (*n* = 80) and Insulin T2DM (*n* = 35) Hospital diabetes clinic.	Questionnaire administered by HCPs

Key: *DSN* diabetes specialist nurse, *PN* practice nurse, *GP* general practitioner, *HCP* health care professional, *OHAs* oral hypoglycaemic agents, *PCPs* primary care physicians, *QOL* quality of life, *SMBG* self-monitoring of blood glucose, *T1DM* type 1 diabetes mellitus, *T2DM* type 2 diabetes mellitus, *Insulin T2DM* insulin treated type 2 diabetes mellitus

In total, 12 themes with 46 sub-themes from the patient studies; and 14 themes with 54 sub-themes from the HCP studies were included in the primary thematic frameworks.

### Integrated themes

**Fig. 2:**
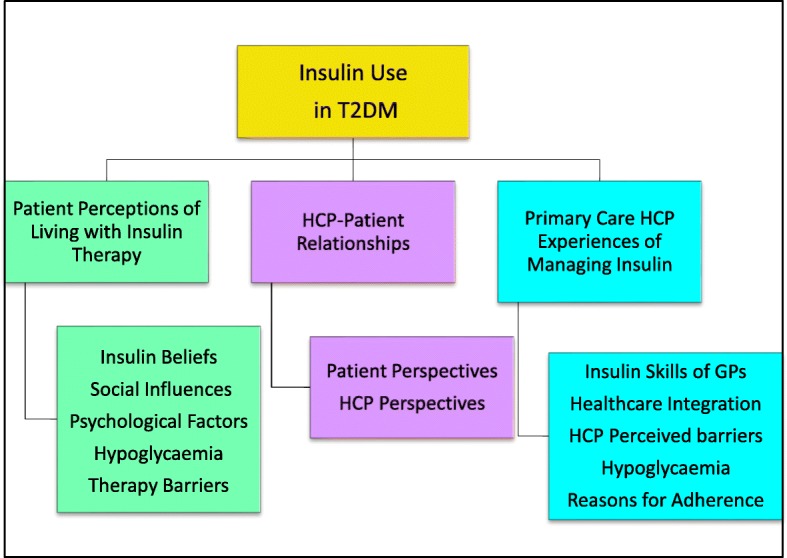
Twelve Primary Themes formed from the Thematic Frameworks. Key: HCP = Healthcare Professional; T2DM = type 2 diabetes mellitus

The synthesis integrated the two thematic frameworks to form 12 primary themes expressed in three domains: patient perceptions, HCP perceptions, and HCP-patient relationships (see Fig. 2.). The themes for each domain are described below with linkage to the source data from qualitative studies (with participant comments) and surveys (which are identified). A summary of the survey findings will then follow. There were more themes relating to barriers than to facilitators to managing insulin.

### Domain 1. Patient perceptions

In this domain five themes relating to patient perceptions of insulin emerged from the synthesis: insulin-related beliefs, social influences, psychological factors, hypoglycaemia, and therapy barriers.

#### Theme 1. Insulin-related beliefs

The data showed that a patient’s beliefs about insulin can mediate their orientation to using insulin. These beliefs include: illness severity; cultural beliefs; and insulin specific beliefs. Many patients reported how when insulin was first suggested, they believed it meant their diabetes had suddenly become very serious [36, 38, 41, 62].

*“…I felt like once you hit insulin you are on a slide to … you know [death].”* [Participant 13] [41]

Survey respondents also reported their perceived seriousness of the condition [62].

Cultural beliefs can influence insulin adherence negatively, particularly when cultural traditions conflicted with the underlying constructions about what insulin was and how diabetes should be treated [36, 38, 41]. One patient from a UK African-Caribbean community said:

*“I’m telling you I’ve known people take insulin here and they go back to the Caribbean and don’t take insulin.… they don’t have the pollution that you have here, your body perspires more so all the impurities or all the stuff that it retains in your body keeps coming out ..”* [Interview 16] [38]

Janes et al. described how cultural beliefs could be in direct conflict with using drugs [41]. One individual relied on traditional Maori beliefs and medicinal plants for healing:

*“The body is tapu [restricted]… it makes me not like poking holes in it* [with needles]*”* [Participant 13]

#### Theme 2. Social influences

Social factors included stigma, family and friends, economics, work and social activities. Perceived stigma relating to injecting in public was associated with insulin adherence [36, 39, 41–43, 59]. For some this stigma was reflected in the belief that others perceived injecting insulin as being associated with drug addiction [36, 41, 42].

*“Our society is quite ignorant of insulin therapy and they might associate insulin injection with drug addicts”* [2 years of insulin use/ 5 years of having diabetes] [36]

Of the T2DM patients (*n* = 27) in Mehmet et al.’s survey [59] the majority (*n* = 20) also experienced problems injecting in public, the main reason being worry about upsetting or offending others.

Patients developed various strategies to adjust for this stigma adding to the complexity of insulin use:

*“If I go out with anybody I always go and do it (inject) in the toilet. I won’t ever do it outside.”* [Participant 26] [42]

Patients were influenced by family and friends in managing their insulin [36, 41, 43, 44]. For some this created barriers to insulin use, as they had to observe the requirements and routines of the family over mealtimes impacting on their insulin behaviours. However, others identified the potentially positive influence of family support [36]:

*“I always refer to these two ‘specialists’ (my father and older brother who are on insulin) when it comes to insulin”* [6 years of insulin use/ 10 years of having diabetes]

*“I gained a lot of knowledge from self-reading and relatives who are on insulin”* [2 years of insulin use/ 5 years of having diabetes]

Economic factors (such as the cost of blood-testing strips and loss of earnings) and employment disruption were both identified as socially specific mediators of insulin use [41, 43, 44]:

*“Cost is a problem. If I went to the doctor plus medication, that was my week’s pay gone.”* [Participant 15] [41]

*“I would come off an ‘18 hour’ and the day shift boss would ring me up, says ‘hey, can you come in and do a couple of hours, bro.’… Insulin was not easy to take and you would pop it in, but no, I had to wait between shifts..”* [Participant 11] [41]

Others, however, felt supported at work [36].

The impact of insulin use on travel, leisure, and social activities was perceived negatively by patients [36, 38–41], as it restricted their social interactions and influenced their insulin injecting behaviours when in social settings:

*“I wouldn’t go out to lunch with them [friends] and in the end, I had to tell them why. I said, ‘I can’t. I have got to have insulin. And I am not going to go into a toilet’.”* [Participant 23] [42]

#### Theme 3. Psychological factors

Psychological factors related to fear and anxiety, shame and depression. Fear and anxiety about hypoglycaemia (see Theme 4), injection pain, and weight gain were perceived by many participants as significant mediators in insulin utilisation [36–38, 40, 41, 43, 44] and included survey participants [53, 61, 65, 69].

*“I am scared of needle.. you know, the poking itself, it is painful.. using needle some more, and you poke yourself... it is painful”* [3 years of insulin use/ 6 years of having diabetes] [36]

Feelings of shame and self-blame were evident in the participant accounts of some studies [38, 41]. These feelings were linked to the perceptions that they had somehow caused their disease and that their need for insulin was because they had not properly controlled their diabetes:

*A good diabetic is one who controls their diabetes …I am not a good diabetic.* [Participant 7] [41]

Negative emotions such depression also had an impact on insulin use. In one large survey depression was the strongest predictor of the severity of fear of self-injecting [61]. In the qualitative studies, negative emotions were often identified in the context of low patient activation in relation to self-management:

*“In that period of depression I was just happy when I felt good and that things were moving again, and that I could do my job again …and for me that was enough. The diabetes just wasn’t that important for me.”* [Participant] [40]

#### Theme 4. Hypoglycaemia

Hypoglycaemia was identified in survey participants as a key barrier and concern for patients with impact on their emotional state, daily functioning and engagement with their insulin [45, 49, 51, 52, 55–58, 60, 66]. In consequence patients reported injecting smaller doses to keep their blood glucose elevated. The survey studies identified that a fear of hypoglycaemia is common and is associated with reduced adherence [49, 51, 57]. The patient accounts in the qualitative studies gave many examples of these behavioural responses to hypoglycaemia:

*“When I am hypoglycaemic, I feel wretched. ... I don’t really have a problem with high sugar levels, but the low ones are quite bothersome.”* [Participant] [40]

*“to avoid hypos… I won’t have my insulin”* [Participant 4] [41]

#### Theme 5. Therapy barriers

The inherent complexities of managing insulin, was often an impediment to insulin adherence in several surveys [48, 50, 62, 63] which reported associations between insulin non-adherence and practical barriers, injection difficulties and regimen inflexibility. Patients remembering whether they had taken their insulin was another factor, with people omitting injections if they were unsure whether they had taken it or not:

*“I am type 2 and when I forget my insulin in the morning, then I skip it and take my next insulin with my next meal.”* [Germany, Male] [37]

The challenges associated with sustaining regular self-monitoring of blood glucose (SMBG) were also identified as impeding insulin behaviours [40, 43]:

*“Beginning [SMBG] yes, beginning very keen, now no. I’m just simply lazy to do it.”* [P06, 69-year-old female retiree, diabetes for 15 years] [43]

While some patients found SMBG to be helpful in achieving better glycaemic control and in detecting hypoglycaemia, others perceived it as a burden [40]. Some patients reduced SMBG once they established a dose they felt was right for them, such that they could not monitor any changes in their insulin requirement [43].

A further area of therapy complexity was in the titration of the insulin dose. Five qualitative studies [37, 40, 42–44] and several surveys [50, 52, 53, 58], reported that patients struggled with titration, often ignoring instructions, or adopting their own approach. There was some divergence between patients as to whether they wanted the HCP to make insulin changes or whether they preferred to control it themselves:

*“I never change the therapy my doctor prescribed! I trust him, that’s his job, not mine!”* [67-year-old woman] [44]

One patient became more confident after receiving appropriate HCP support:

*“At first I was very afraid about changing my dosage of insulin. But then my doctor explained to me how... In the beginning, I used to call him, but now I frequently change the dosage on the basis of my own physical activity, diet, and sugar levels.”* [55-year-old woman] [44]

Whilst a majority of patients and physicians regarded insulin therapy as restrictive in one survey [64], more patients saw insulin treatment as having positive than negative impacts on their life though this trend was less in T2DM than T1DM individuals.

### Domain 2. HCP perceptions

Five themes emerged in relation to HCP perceptions: insulin-related skills of GPs, healthcare integration, HCP perceptions of patient-related barriers, hypoglycaemia, and HCP explanations for insulin adherence.

#### Theme 1. Insulin-related skills

This theme relates to the skills required by primary care HCPs to initiate and intensify insulin therapy, and to provide ongoing support for patients. While many HCPs were positive about helping patients to manage insulin, others felt they lacked the skills to do so effectively [45, 46]. They believed insulin-related training was important, but they also wanted ongoing support from a diabetes specialist. GP attitudes seemed to modify when they had acquired insulin-related skills, increasing their motivation and confidence in supporting patients:

*“My attitude about insulin therapy onset has changed. Before the start of 0f the project, I tried too long oral anti diabetics, but the courses have changed my attitude. I became confident in starting insulin therapy, whereas before I would never initiate insulin therapy.”* [GP12-S3] [45]

However, some felt excluded, believing that specialists wanted to continue to manage insulin treated patients themselves:

*“Specialists gain too much control of referred patients and often exclude GPs from direct patient care. This is especially true of patients on insulin who get free instructions and monitoring kits at the diabetes centres, unlike patients in primary care. So, it's nearly impossible for GPs to hold on to patients on insulin.”* [GP1-S2] [45]

In one survey [54], there was disagreement regarding who was responsible for intensification but the majority of both diabetes specialists and primary care physicians agreed that doctors in primary care should become more involved in managing insulin. In another [67] nurses and physicians agree that nurses should take a larger role in managing diabetes.

#### Theme 2. Healthcare integration

The level of integration between the different components of the health system was identified as having a key role in how patients were supported in using insulin [45, 47] as illustrated by this GP:

*“This is a big change from the usual 'let us do our work; after all we are the specialists and you may help a little bit'. We collaborate as one team – there's mutual support! We're on the same wavelength and feel we work together toward the same objectives.”* [GP13-S4] [45]

Better collaboration between primary and secondary care was considered by most physicians in Cuddihy et al.’s survey [57] as one of the most important factors in improving insulin treatment of T2DM.

The systems in which primary care HCPs work influenced how involved they are in starting and/or managing insulin therapy. GPs and PNs identified that a lack of resources and familiarity with starting and managing insulin impacted negatively on the insulin support they could provide [46]. One large Dutch survey observed that the more structured practices employing a PN and with a designated diabetes clinic were more likely to manage insulin therapy themselves [68].

#### Theme 3. HCP perceptions of patient barriers

HCPs reported that patient-level factors heavily influenced insulin use, echoing many of those voiced by the patients, including: beliefs, culture, economics and psychological barriers. In addition, they believed patient education impacted positively on insulin use [45–47]. They felt, that for patients, insulin treatment represented failure and a more serious stage of the illness:

*“I think probably they think it’s the end, that’s it, there’s nothing else they can have after that.”* [HCP] [46]

They also identified that patients often altered their insulin behaviours subjectively based on how they felt, rather than by following their targets [47]. Some found it challenging when dealing with patients from different ethnic backgrounds [46]. They reported how some cultural beliefs created barriers to insulin use:

*“We see patients twice a year and the family and friends are there all the time, you know, I mean, we are supposed to be more powerful figures, but I mean, it’s quite difficult to overcome very different beliefs within the family.”* [HCP] [46]

HCPs perceived that SMBG for insulin optimisation was moderated by fear and in some countries cost:

*“Those who can afford also don’t see that it’s important to invest on the glucometer … When we talk about meter and everything, you have to talk about fear of pricking. That’s another barrier.”* [Family medicine specialist, public health clinic] [47]

*“How come when we [public health clinics] give all [insulin and pens], we provide everything free, but the glucometer is not given, test strips are not given, and how are they [patients] monitoring the blood glucose?”* [GP, private general practice] [47]

The psychological factors identified by the HCPs again reflected the insulin-related fears and anxieties reported by patients, such as: hypoglycaemia, concerns about weight gain, and fear of injection pain.

*“Surely, one of the biggest barriers is this fear of going onto needles for the rest of your life. I think the effect of getting older is that they hate the idea of hypoglycaemia as well. They get very frightened of that.”* [HCP] [46]

HCPs believed patients had insufficient understanding of diabetes and needed much more input in relation to insulin titration and dose adjustment if they were going to use insulin effectively [46, 47]:

*“So … the most common thing, what happen is, people start insulin, but after that, they don’t optimize and specify the regime. The patient who started just on one regime for, like, many years and nobody have actually taught the patient how to do the self-titration of the insulin too* ….” [Family medicine specialist, public health clinic] [47]

#### Theme 4. Hypoglycaemia

HCPs identified fear of hypoglycaemia as a significant issue in optimal insulin use in surveys [54, 58, 64] and interviews [46, 47].

*“I think the effect of getting older is that they hate the idea of hypoglycaemia as well. They get very frightened of that.”* [HCP] [46]

#### Theme 5. Explanations for insulin adherence

HCP explanations for low insulin adherence included: being too busy; travelling; the timing of meals; stress or emotional problems; public embarrassment; and the patient’s perception of their diabetes control:

*“….so it depends how their [patients’] lifestyle... It depends on their work also … how’s their working and meal times. Their mealtimes also … they will tell us.”* [Family medicine specialist, public health clinic] [47]

*“Maybe they [patients] will continue [using insulin] for a while, they will get better, they said, No, I don’t want injection anymore.”* [R1, GP, private general practice] [47]

*“They said ‘I am better, so I can stop now.’”* [R2, GP, private general practice] [47]

HCPs in surveys [50, 64] reported that their typical patient did not take their insulin as prescribed citing similar reasons as patients [64]. Prescribers did not routinely discuss basal adherence patterns with their basal-bolus patients [50].

### Domain 3. HCP-patient relationships

This domain identifies the role of the HCP-Patient relationship, with regard to insulin therapy utilisation. For patients, communication and relational care were important in shaping their insulin views and behaviours. From the HCPs perspective, their interactions with patients were influenced by their personal confidence in using insulin therapy. The domain is comprised of two themes.

#### Theme 1. Patient perspectives of relational care

The quality of the relationship and communication with HCPs was valued by patients. In many of the qualitative studies it was identified as an important factor contributing to their adherence to insulin [36, 38, 40, 44] and in surveys [52, 62]. The nature of the relationship could contribute positively or negatively on the patient’s insulin behaviours depending. Key factors that influenced the quality of the relationship were: how the HCP communicated insulin-related information; whether they elicited and responded to patient concerns; the time available for the consultation; and how accessible and relevant the support provided was to the patient:

*“I have got a good doctor… but they are busy, real busy, and I suppose you have not got time to talk.”* [Patient 8] [41]

*“..we discussed about the issues of insulin, my worries and thoughts about insulin. I became less apprehensive and was ready to start on insulin therapy”* [2 years of insulin use/ 5 years of having diabetes] [36]

Another aspect of the relationship was reflected in the divergent agenda of the HCP and the patient. While HCPs tended to focus on tightening glycaemic control, patients were more concerned with their wider life needs and their quality of life (QOL). This was reflected in the ways patients moderated their behaviour to try and appease the HCPs:

*“I have been using it [SMBG] every day because I know I have got an appointment coming up, so I better behave [participant giggled]. So that I can tell the doctor, you know, I want to bring down the insulin dose.”* [P01, 57-year-old female clerk] [43]

#### Theme 2. HCP perspectives

HCP perceptions of their relationship with patients included the impact of integrated care working, the time available for providing insulin-related support, their own ambivalence about insulin therapy, and whether they had the required skills [45–47] and included surveyed HCPs [67, 68]. It was perceived that the relationship between GPs and patients was enhanced when the GPs were equipped with insulin-related skills with good support from diabetes specialist services:

*“Diabetes patients themselves feel much more appreciated; because of that, the link between us and our patients has strengthened.”* [GP17-S4] [45]

When the HCP adopted a patient-centred approach in their relationship, this could enhance insulin use:

*“…Because when we negotiate, you know, some, they said okay, after negotiating, then they’re okay. Then they try to follow.”* [Family medicine specialist] [47]

### Summary of the survey findings

The surveys reported a number of factors that might mediate insulin use. In the patient based surveys (*n* = 14) these included: hypoglycaemia [49, 52, 55–57, 60, 66] glycaemic control [66], injecting in public [59], problems with injections [61, 69], insulin intensification [53], insulin adherence [48, 63], and perceptions of T2DM [62]. Studies with both patients and HCPs (*n* = 5), identified hypoglycaemia [49, 58], dosing irregularities [50, 58], insulin adherence [64], and injection-related problems [65]. HCP surveys (*n* = 3) included: insulin intensification [54], HCP perceptions of nurse involvement in T2DM [67], and insulin management in general practice [68]. A table of the findings and key topics can be viewed in Additional file 3.

#### Patient-related themes

##### Hypoglycaemia

Hypoglycaemic events associated with insulin, particularly nocturnal hypoglycaemia, were reported as having a disrupting effect on: diabetes self-management; sleep quality and next-day functioning; work performance and driving; and personal well-being [49, 52]. It was also reported that many patients with T2DM had no warning signs of hypoglycaemia [55]. Some studies reported that hypoglycaemia had negative financial consequences and impact on QOL [49, 56, 66]. Severe hypoglycaemic episodes led people to fear future events [57] with subsequent worse self-reported glycaemic control [60]. Exposure to insulin-related hypoglycaemia was reported to lead to poor insulin adherence and omission [48, 49, 58, 63, 66].

##### Injection-related problems

Patients in Mehmet et al.’s [59] study reported problems injecting in front of others, most commonly because they worried about upsetting or offending them. Others experienced anxiety and fear of injections [61]. Zambanini et al. [69] found insulin injections were avoided in 14% of participants because of related anxiety.

##### Adherence to insulin

Insulin non-adherence was common taking the form of dosing irregularities and insulin omission [50, 58, 64, 65]. Factors contributing to insulin adherence, included: being in a public place or travelling; fear of hypoglycaemia; and therapy complexity [48, 63].

The majority of patients surveyed by Cefalu et al. [53]; wished there was another way to take insulin whether they were using insulin (*n* = 371;79%) or not (*n* = 782; 80%). Non-adherence was also associated with dosing irregularities, reduced doses, and mistimed doses [50, 58, 64, 65].

#### HCP-related themes

##### Adherence to insulin

Physicians (55% from primary care and 45% specialists) reported that glucose control was negatively impacted by the level of insulin adherence, with missed, mistimed, or reduced insulin doses being identified [50]. Despite acknowledging the clinical relevance of irregular dosing, 32% of physicians reported not routinely discussing these with their basal insulin patients and 29% with their basal-bolus patients.

Hypoglycaemia was identified by HCPs as having an effect on insulin adherence [49, 58]. In Peyrot et al.’s study [64], patients and physicians agreed the five most common reasons for insulin omission or non-adherence was being too busy; travelling; skipped meals; stress or emotional problems; and public embarrassment. Rubin et al. [65] reported 50% of their patients would be more likely to take insulin regularly if the pain of injecting could be ameliorated.

##### Insulin-related role

Cuddihy et al. [54] surveyed 600 physicians (50% from primary care and 50% specialists) and found that notable proportions of primary care physicians never initiate or modify insulin and never or rarely intensify it mainly because of lack of experience and lack of time to educate patients. There was also disagreement regarding who was responsible for intensification. However, 86% of all the physicians agreed that primary care physicians should become more involved in managing insulin. In another study [67] nurses and physicians agreed, nurses should take a larger role in managing diabetes. Finally, in a survey by Van Avendonk et al. [68] of Dutch GPs (*n* = 1621) 67% started and managed insulin therapy in T2DM. Associated factors were being male, above age 40 years, working in a health centre, and working together with a Practice Nurse.

### Analytical themes

**Fig. 3:**
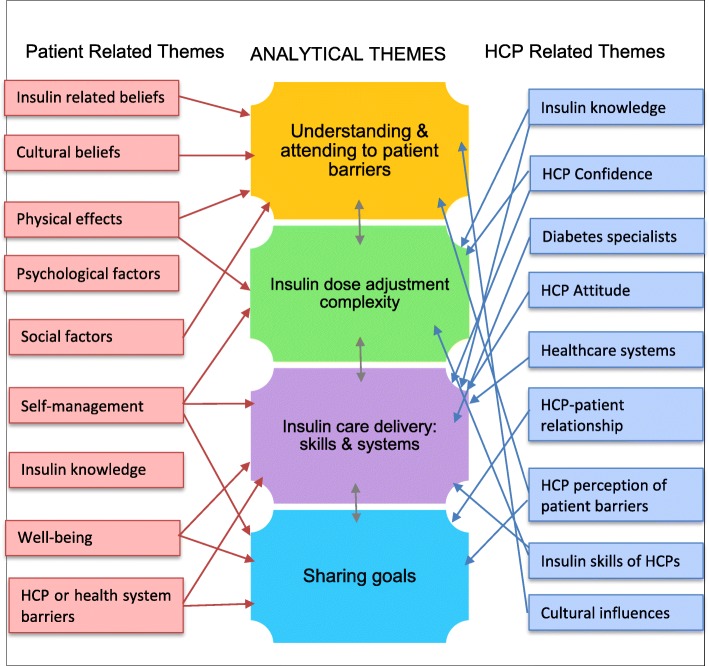
Analytical Model and the Interrelated Themes. Key: HCP = Healthcare Professional

Four analytical themes, the equivalent of third-order interpretations in meta-ethnography [19, 26], were then generated from the integrated themes. These interpretations provided new perspectives to identify modifiable mechanisms that could be manipulated to enhance insulin use and adherence. The themes are interrelated as expressed in the model outlined in Fig. 3.

#### Theme 1. Understanding and attending to patient barriers

It is evident that there are multiple barriers to insulin uptake and utilisation in patients with T2DM. These barriers are common and are multi-levelled, with major factors being: psychological issues such as fear or hypoglycaemia and negative beliefs about insulin; and social factors such as external prejudice, stigma and life disruption/constraints. Despite being aware of these patient level barriers to insulin adherence, the primary care HCP accounts did not identify strategies for addressing them. If these barriers are to be overcome a multi-modal approach providing targeted support to patients and enhancing the primary care HCP’s skills in overcoming these are required, with key components being: patient centred education and self-management support addressing patient level barriers; training for primary care HCPs to enhance their confidence in using insulin and in being able to elicit and respond to patient needs in relation to insulin self-management.

#### Theme 2. Insulin dose adjustment complexity

The collected data suggests that current methods of insulin dose titration are not always systematic but are often suboptimal with poor adherence. Dose adjustment seems to be further complicated by patient perceptions on insulin use which can be subjective and are influenced by factors such as avoiding hypoglycaemia and the management of wider aspects of their social and working lives. Hence, if insulin dose adjustment is to be more optimally managed then there is a need for a simpler patient centred approach. This approach again needs to attend to potential behavioural confounders and ensure that patients have a clear perspective on the process, its importance and what they hope to achieve.

#### Theme 3. Sharing goals

The data identified that there may be some divergence between the patient’s blood glucose goals and those of their HCPs. Patients identified that HCPs focussed more on achieving a glycaemic target whereas subjectively they may feel better with higher glucose levels. Hence, insulin use may be enhanced if there is a stronger connectivity between the patient and the HCP in setting and agreeing therapy goals.

#### Theme 4. Insulin care delivery: Skills and systems

The insulin-related skills and attitudes of primary care HCPs may be significant in determining the use of insulin and the outcomes achieved. The skills are not isolated to the individual HCP, as the data suggest that the context of practice is important too, placing an emphasis on systemic factors including care integration and teamwork. This emphasis is further reinforced by the data highlighting continuity and consistency in the support provided to patients. There were also data suggesting the need to integrate specialist support within the system to help primary care professionals optimise care delivery. Where available, the specialist support could also be provided by those practices already experienced and skilled in insulin initiation/intensification. Therefore, if insulin therapy is going to be better delivered within primary care not only will the HCPs need better training, they will also need to develop support systems that are internally (a team approach) and externally integrated (specialist support).

## Discussion

This synthesis has identified a wide range of factors that modify the use of insulin in people with T2DM. These factors can be broadly divided into three interrelated levels, the patient, the HCP and the care system. The use of data derived from both patients and HCPs enhances the analytical potential of the synthesis to consider the interactive components expressed from these different perspectives. These generated the potential development of newer services for patient benefit.

### Patients

The findings of the review have identified a wide range of factors that drive patients’ behaviours in relation to insulin use. These factors include: underlying beliefs about insulin; psychosocial factors; the self-management skills and knowledge of the patient; and their experiences in using insulin. It is also clear from the review that many of these factors are interactive. While many of these factors have been reported in previous reviews [10, 13, 16, 70] this review has considered how these factors are expressed and interact in the experiences of patients with the added perspective of how they relate to the views and behaviours of health professionals. This latter element is important as it is the interaction between patients and health professionals where many of the challenges and barriers for effective insulin use in patients with T2DM reside. The review has also highlighted the problems and issues that affect patients’ use of insulin. Addressing these issues is important and they need to be considered in the patient education and self-management support provided to patients. The findings suggest that as well as the technical aspect of self-management, the support provided needs to consider the patients’ underlying beliefs, their psychological orientation to insulin and the influence of wider social factors. Addressing the problem of clinical and psychological inertia of the intensification of insulin therapy is key part of the process [8, 12, 71]. Given that factors such as perceived stigma in their use of insulin restricts how they use it, it may be important to help patients develop strategies to ameliorate those feelings. Wider factors such as family dynamics also need to be considered. Therefore, if patients are going to supported in using insulin effectively the barriers and factors highlighted in the review need to be incorporated into the insulin assessment process and attended to in the self-management support provided. It is also necessary to establish whether or not a patient wishes or is able to self-manage their insulin titration as some patients may prefer to be led by their HCP as was apparent in this synthesis [42, 44],

### HCPs

The HCPs accounts utilised within the review were predominantly those of primary care physicians. While these accounts were derived from studies undertaken in different healthcare systems, they shared similar perspectives on insulin management. The two key factors that governed the delivery of insulin care were the skills of the HCP and the time available. The former would suggest that there is a need for professional education. Given the findings of the patient accounts, this education needs to offer more than the technical aspects of insulin and should include an understanding of the psychosocial factors that may influence insulin use. In relation to time, it may be important to identify the role of other team members in delivering insulin support such as primary care nurses or diabetes specialist nurses supporting the primary care team. These benefits were highlighted in a study by Ritholz et al. [72] but the physician participants stressed the necessity of regular and ongoing communication among team members to ensure patients received consistent information. The review has also identified that the interactions between HCPs and patients are pivotal in determining whether insulin is used effectively. The relational aspect of care and continuity of support seem to be particularly important. In keeping with other studies [73, 74], the review identified that patients and HCPs can sometimes have divergent views in some areas, in particular glycaemic targets, highlighting the need for agreeing blood glucose goals in a collaborative way when supporting patents to adjust their insulin. Serrano et al. [75] illustrated some effective approaches to shared-decision making to enhance patient understanding of choices in diabetes management. Therefore, primary care HCPs can have a very important contribution to make in using insulin effectively provided they have the appropriate training, the time needed to deliver care and supportive health systems.

### Healthcare systems

The evidence presented in this thematic synthesis revealed how integrated healthcare systems, teamwork, the way GP practices were organised, and in one study, the presence of a Practice Nurse [68], all facilitated the role of general practice in insulin treated T2DM. Diabetes specialists also shared this view. The thematic synthesis identified that the support of diabetes specialist teams, can help primary care HCPs to deliver insulin support. Therefore, to ensure that insulin is used optimally in primary care, the findings of the review indicate that the care system needs to be designed to ensure that patients are assessed and followed up by an appropriately trained HCP, who can provide continuity in their care experience. The system also needs to consider how to integrate specialist diabetes support to help the primary care teams in their clinical decision making and in building the resources that patients will need to support their insulin use.

### Limitations

This review has a number of limitations. The principle one is the reliance on the quality of the data from the primary studies, as most of the studies were not exclusive to T2DM patients, and not all based only in primary care. The latter was addressed by only including data from participants with T2DM in primary care. Another limitation was that many of the studies were biased toward the perspectives of primary care physicians, and identifying more accounts from other team members would have enhanced the review findings. From a UK perspective, more accounts of the contribution from PNs would have been desirable. It was also noted that while it was possible to elicit barriers to effective insulin utilisation, there were few studies that identified potential facilitators of insulin management, although the review was able to theorise these based on the nature of the identified barriers. Another potential source of bias was that some of the surveys were supported by insulin-related companies, although no evidence of such bias and the nature of the surveys were not related to product evaluation. The inclusion of both qualitative and quantitative designed studies is a further weakness, particularly with the variety of qualitative approaches, incorporating interpretive and descriptive approaches. However, this integration could also be viewed as a strength as identifying common themes in the different data sources adds to the likely generalisability of the findings. Finally, the literature search was completed in March 2015 and further studies have since been identified. These include a patient survey of frequency of self-treated hypoglycaemia [76], a focus group study of insulin treated T2DM patients to identify fear of hypoglycaemia [77], interviews and focus groups of patients and HCPs to ascertain their perspectives on psychological insulin resistance [78], semi-structured interviews of patients to establish barriers to and enablers of insulin self-titration [79], and finally interviews of patients with insulin treated T2DM to detect their reasons for poor glycaemic control [80].

Despite these limitations, the synthesis has provided some novel insights into the collective factors impacting on insulin treated patients in primary care. These will be a helpful reference for further exploratory studies in developing new interventions.

## Conclusions

Insulin use is often poor in people with T2DM, and associated with sub-optimal long-glycaemic control, with risk of complications and increased mortality [10–12, 81, 82]. This review reveals the burden experienced by T2DM patients receiving insulin and the skills needed to equip primary care HCPs to support them. Integrated healthcare systems with appropriate resources could help facilitate this but patient-centred care by appropriately skilled GPs and PNs is also required.

## Additional files

Additional file 1: Literature Search Strategy (DOCX 54 kb)

Additional file 2: Appraisal scores for the included studies. (DOCX 23 kb)

Additional file 3: Findings of the Surveys. (DOCX 29 kb)

## Abbreviations

CASP: Critical appraisal skills programme
DSN: Diabetes Specialist Nurse
GP: General Practitioner
HCP: Healthcare professional
OHAs: Oral hypoglycaemic agents
PCP: Primary Care Physician
PN: Practice Nurse
QOL: Quality of life
RR: Response-rate
SMBG: Self-monitoring of blood glucose
T1DM: Type 1 diabetes mellitus
T2DM: Type 2 diabetes mellitus
