# Supplementary material for: Perceptions of insulin use in type 2 diabetes in primary care: a thematic synthesis
Source: BMC Fam Pract. 2018 May 22;19:70. doi: 10.1186/s12875-018-0753-2 (PMC5964885; doi:10.1186/s12875-018-0753-2)
Supplement: Supplementary file 1 — Literature Search Strategy (DOCX 54 kb) [file 12875_2018_753_MOESM1_ESM.docx]

Literature Search Strategy: Electronic Data Bases

| CINAHL   1. Diabetes Mellitus, Type 2 2. Insulin 3. S1 and S2 4. Patients or “patients” 5. Advanced Practice Nurses or “practice nurses” 6. Nurses or “nurses” 7. Physicians or “doctors” 8. “general practitioners” 9. “health care professional” 10. S4 or S5 or S6 or S7 or S8 or S9 11. S3 and S10 12. Perception or “perception” 13. “experiences” 14. Health Behaviour or “health behaviour” 15. Health Beliefs 16. Patient Compliance or “adherence” 17. S12 or S13 or S14 or S15 or S16 18. S11 and S17   COCHRANE LIBRARY   1. Diabetes Mellitus, Type 2 2. Insulin 3. #1 and #2 4. “insulin treated type 2 diabetes” 5. #3 or #4 6. Patients 7. Nurses 8. “practice nurse* 9. General Practitioners 10. “general practitioner* 11. Patient Care Team 12. “health care professional* or “patients” 13. #6 or #7 or #8 or #9 or #10 or #11 or #12 14. #5 and #13   EMBASE   1. exp non-insulin dependent diabetes mellitus/ or “insulin treated type 2 diabetes” 2. exp insulin/ 3. 1 and 2 4. exp patient/ or “patients” 5. exp health care personnel/ or “health care professional*” or exp physician/ or exp nurse/ 6. 4 or 5 7. 3 and 6 8. exp perception/ or “perception*” 9. exp experience/ or exp personal experience/ or “experience* 10. health behaviour/ or attitude to health/ or patient compliance/ or health belief/ or “health belief*” 11. “understand*” or “adherence” or “concordance” 12. 8 or 9 or 10 or 11 13. 7 and 12 14. exp primary medical care/ or “primary care” 15. exp general practice/ or “general practice” 16. 14 or 15 17. 13 and 16   MEDLINE   1. exp Diabetes Mellitus, Type 2/ or “insulin treated type 2 diabetes” 2. exp Insulin/ 3. 1 and 2 4. Patients/ or “patient” 5. exp Delivery of Health Care/ or “health care professional*” or exp Physicians/ 6. exp Physicians, Family/ or exp General Practitioners/ or “general practitioner*” 7. exp Nurses/ or “nurse” 8. exp Nurse Practitioners/ or “practice nurse*” or exp Nursing Staff/ 9. 4 or 5 or 6 or 7 or 8 10. 3 and 9 11. exp Perception/ or “perception*” 12. exp Attitude to Health/ or exp Health Behaviour/ or “health behaviour” 13. exp Compliance/ or Patient Compliance/ or “compliance” 14. exp Medication Adherence/ or Patient Compliance/ or “adherence” 15. “understand*” or “experience*” or “concord*” 16. 11 or 12 or 13 or 14 or 15 17. 10 and 16 18. exp Family Practice/ or General Practice/ or “general practice” 19. exp Primary Health Care/ or “primary care” 20. 18 or 19 21. 17 and 20   PsycINFO   1. exp Diabetes Mellitus/ or “insulin treated type 2 diabetes” 2. exp Insulin/ or “insulin” 3. 1and 2 4. exp Nurses/ or “practice nurse* or exp General Practitioners/ 5. exp Physicians/ or “doctor*” 6. exp Health Personnel/ or exp Health Care Delivery/ or “health care professional*” 7. patient selection/ 8. exp Patient Selection/ or “patient*” 9. 4 or 5 or 6 or 7 or 8 10. 3 and 9 11. “experience*” or “perception*” 12. exp Health Behaviour/or exp Behaviour Change/ or exp Health Attitudes/ or “health behaviour” 13. exp Treatment Compliance/ or “adherence” 14. 11 or 12 or 13 15. 10 and 14   WEB OF SCIENCE   1. Type 2 Diabetes and Insulin 2. “experience*” or “perception*” or “understand*” 3. #2 and #1 4. “primary care” or “general practice” or “community” 5. #4 and #3 |
| --- |

| **EMBASE Search conducted 20 October 2014**   1. exp non-insulin dependent diabetes mellitus/ or “insulin treated type 2 diabetes” 2. exp insulin/ 3. 1 and 2 4. exp patient/ or “patients” 5. exp health care personnel/ or “health care professional*” or exp physician/ or exp nurse/ 6. 4 or 5 7. 3 and 6 8. exp perception/ or “perception*” 9. exp experience/ or exp personal experience/ or “experience* 10. health behaviour/ or attitude to health/ or patient compliance/ or health belief/ or “health belief*” 11. “understand*” or “adherence” or “concordance” 12. 8 or 9 or 10 or 11 13. 7 and 12 14. exp primary medical care/ or “primary care” 15. exp general practice/ or “general practice” 16. 14 or 15 17. 13 and 16 | **Results**  147873  272943  40864  6062479  1012339  6717071  21230  398090  1008881  219457  929472  2343077  2966  113451  86163  186276  215 |
| --- | --- |
