# Supplementary material for: Perceptions of insulin use in type 2 diabetes in primary care: a thematic synthesis
Source: BMC Fam Pract. 2018 May 22;19:70. doi: 10.1186/s12875-018-0753-2 (PMC5964885; doi:10.1186/s12875-018-0753-2)
Supplement: Supplementary file 2 — Appraisal scores for the included studies. (DOCX 23 kb) [file 12875_2018_753_MOESM2_ESM.docx]

APPRAISAL OF THE INCLUDED STUDIES

Appraisal of the Qualitative Studies using the CASP Qualitative Research Checklist [37].

| First Author (year)  Score | Q1 Was there a clear statement of the aims of the research? | Q2 Is a qualitative methodology appropriate? | Q3 Was the research design appropriate to address the aims of the research? | Q4 Was the recruitment strategy appropriate to the aims of the research? | Q5 Was the data collected in a way that addressed the research issue? | Q6 Has the relationship between researcher and participants been adequately considered? | Q7 Have ethical issues been taken into consideration? | Q8 Was the data analysis sufficiently rigorous? | Q9 Is there a clear statement of findings? | Q10 How valuable is the research? |
| --- | --- | --- | --- | --- | --- | --- | --- | --- | --- | --- |
| Abu Hassan (2013)  10/10 | 1 | 1 | 1 | 1 | 1 | 1 | 1 | 1 | 1 | 1 |
| Brod (2014)  8/10 | 1 | 1 | 1 | 1 | 1 | 0 | 1 | 0 | 1 | 1 |
| Brown (2007)  10/10 | 1 | 1 | 1 | 1 | 1 | 1 | 1 | 1 | 1 | 1 |
| Browne (2013)  10/10 | 1 | 1 | 1 | 1 | 1 | 1 | 1 | 1 | 1 | 1 |
| Goderis (2009)  9/10 | 1 | 1 | 1 | 1 | 1 | 1 | 0 | 1 | 1 | 1 |
| Hortensius (2012)  9/10 | 1 | 1 | 1 | 1 | 1 | 0 | 1 | 1 | 1 | 1 |
| Janes (2013)  10/10 | 1 | 1 | 1 | 1 | 1 | 1 | 1 | 1 | 1 | 1 |
| Jeavons (2006)  9/10 | 1 | 1 | 1 | 1 | 1 | 1 | 0 | 1 | 1 | 1 |
| Jenkins (2011)  10/10 | 1 | 1 | 1 | 1 | 1 | 1 | 1 | 1 | 1 | 1 |
| Lee (2013)  10/10 | 1 | 1 | 1 | 1 | 1 | 1 | 1 | 1 | 1 | 1 |
| Ong (2014)  10/10 | 1 | 1 | 1 | 1 | 1 | 1 | 1 | 1 | 1 | 1 |
| Vinter-Repalust (2004)  9/10 | 1 | 1 | 1 | 1 | 1 | 0 | 1 | 1 | 1 | 1 |

Key: The score of 1 was given where the study answered most parts of the CASP question.

Appraisal scores for the Quantitative Studies using Barley et al.’s Appraisal Tool [30].

| First Author  (year)  Score | Screening Question: Was there a clear aim? | Q1 Was the selection of the participants appropriate? | Q2  Was the measurement of variables appropriate? | Q3 Was there appropriate control of bias? | Q4 Was the use of statistics appropriate? | Q5 Was the study free of conflict of interest? | Q6 List any other limitations of the study |
| --- | --- | --- | --- | --- | --- | --- | --- |
| Ary (1986)  7/7 | 1 | 1 | 1 | 1 | 1 | 1 | 1 |
| Brod (2012a)  3/7 | 1 | RR Patients: 27.5%  RR HCPs: 14%  0 | 0 | 1 | 1 | 0* | Self-reported hypo data |
| Brod (2012b)  3/7 | 1 | RR Patients: 27.5%  RR HCPs: 14%  0 | 0 | 1 | 1 | 0* | Self-reported dosing irregularities |
| Brod (2012c)  4/7 | 1 | 1 | 0 | 1 | 1 | 0* | Self-reported hypo data |
| Brod (2013)  4/7 | 1 | 1 | 0 | 1 | 1 | 0* | Self-reported hypo data |
| Cefalu (2008)  4/7 | 1 | 1 | 0 | 0 | 1 | 0* | 1 |
| Cuddihy (2011)  4/7 | 1 | 1 | 0 | 1 | 0 | 0* | 1 |
| Diago-Cabezudo (2013)  4/7 | 1 | 1 | 0 | 0 | 1 | 0* | 1 |
| Fulcher (2014)  4/7 | 1 | 1 | 1 | 1 | 0 | 0* | Self-reported hypo data |
| Leiter (2005)  5/7 | 1 | 1 | 1 | 1 | 1 | 0* | Self-reported hypo data |
| Leiter (2014)  4/7 | 1 | 1 | 0 | 1 | 1 | 0* | Self-reported dosing irregularities |
| Mehmet (2015)  4/7 | 1 | RR !00%  1 | 0 | 1 | 0 | 1 | No information on funding or NHS Ethics |
| Mitchell (2013)  5/7 | 1 | 1 | 1 | 1 | 1 | 0* | Self-reported clinical data |
| Mollema (2001)  6/7 | 1 | RR 49.5%  1 | 1 | 1 | 1 | 1 | Self-reported clinical data |
| Mosnier-Pudar (2009)  6/7 | 1 | RR 77%  1 | 1 | 1 | 1 | 0* | 1 |
| Peyrot (2012a)  4/7 | 1 | 1 | 0 | 1 | 1 | 0* | Self-reported adherence data |
| Peyrot (2012b)  4/7 | 1 | 1 | 0 | 1 | 1 | 0* | Self-reported adherence data |
| Rubin (2009)  4/7 | 1 | 1 | 0 | 1 | 1 | 0* | Self-reported clinical data |
| Shiu (2004)  7/7 | 1 | RR 70%  1 | 1 | 1 | 1 | 1 | 1 |
| Siminerio (2007)  5/7 | 1 | 1 | 0 | 1 | 1 | 0* | 1 |
| Van Avendonk (2009)  5/7 | 1 | RR 42%  1 | 0 | 1 | 1 | 0* | 1 |
| Zambanini (1999)  6/7 | 1 | 1 | 1 | 0 | 1 | 1 | 1 |

For all questions except Question 6, the score of 1 was given where the study answered most of the tool’s question.

For Question 6, 1 = no other limitation.

Key: RR = Response Rate (included if available); HCP = Healthcare Professional

Hypo = hypoglycaemia

*The study was supported and/or funded by a diabetes-related pharmaceutical or medical device company.
