# Supplementary material for: Perceptions of insulin use in type 2 diabetes in primary care: a thematic synthesis
Source: BMC Fam Pract. 2018 May 22;19:70. doi: 10.1186/s12875-018-0753-2 (PMC5964885; doi:10.1186/s12875-018-0753-2)
Supplement: Supplementary file 3 — Findings of the Surveys. (DOCX 29 kb) [file 12875_2018_753_MOESM3_ESM.docx]

SUMMARY OF THE SURVEY FINDINGS

|  | **Topic** | **Author & Reference** | **Aim** | **Participants** | **Summary of the Findings Relevant to Insulin Treated T2DM** |
| --- | --- | --- | --- | --- | --- |
|  |  |  |  |  |  |
| PATIENTS ONLY | Hypoglycaemia | Brod [51] | To determine how non-severe nocturnal hypoglycaemic events (NSNHEs) affect diabetes management, sleep quality, functioning, and to assess if these impacts differ by diabetes type or country. | T1DM and T2DM patients (n=1086) who experienced NSNHE in the last month:  T1DM (n=676)  Non-Insulin T2DM (n=124)  Insulin T2DM (n=286) | - Of 1,086 respondents experiencing ≥1 NSNHE whilst asleep at night, 38.9% (n=422) reported experiencing their most recent NSNHE in the past week, 30.5% in the last 2 weeks, and 30.7% reported experiencing their NSNHE in the last month, but more than 2 weeks prior. - T2DM respondents required significantly more time than T1DM to recognize and respond to the event (1.5 vs 1.1 hours), 25.7% (T1DM), - NSNHEs were associated with a substantial impact on diabetes management, sleep quality, and next-day functioning |
|  | Hypoglycaemia | Brod [54] | To explore the burden and impact of NSNHEs on diabetes management, patient monitoring and well-being to better understand the role NSNHEs play in caring for people with diabetes and to facilitate optimal diabetes treatment strategies. | Patients (n=2,108) with:  T1DM or T2DM.  T1DM (n=692)  Non-insulin T2DM (n=543)  Insulin T2DM (n=873) | - NSNHEs have serious consequences for patients including affecting sleep, next day functioning, driving, and reduced well-being. - Participants were late or absent for work, missed a meeting or work appointment, or had not finished a task on time. - All were likely to take additional SMBG and decreased their insulin dose. |
|  | Hypoglycaemia | Diago-Cabezudo [57] | To evaluate the effects of hypoglycaemia on the lives of patients with DM and determine if SMBG to prevent hypoglycaemic is an appealing and widely accepted concept. | Insulin treated patients (n=1,848)  T1DM (n=924)  Insulin T2DM (n=924) | - Approximately 1/3 of patients were not always able to recognize symptoms of hypoglycaemia when having an episode, about 1/4 had no warning signs. - 37% tended to maintain their blood glucose levels above physician recommended levels to help avoid hypoglycaemia. - 80% said they would value a meter that provides high or low glucose warnings at specific time points during the day. - Overall, people with T1DM and insulin-treated T2DM had a positive perception about and were keen to adopt tools designed to facilitate the identification, management and prevention of hypoglycaemia while helping to avoid hyperglycaemia and an increased risk of diabetic complications. |
|  | Hypoglycaemia | Fulcher [58] | To understand the impact of nocturnal and daytime non-severe hypoglycaemic events on healthcare systems, work productivity & QOL in T1DM or T2DM. | T1DM (n=64)  Non-insulin T2DM (n=76)  Insulin T2DM (n=160) | - Findings suggest nocturnal and daytime non-severed hypoglycaemic events have a large financial and psychosocial impact. - Diabetes management that minimizes hypoglycaemia while maintaining good glycaemic control might positively impact on the psychological wellbeing of people with diabetes, as well as reducing healthcare costs and increasing work productivity. |
|  | Hypoglycaemia | Leiter [59] | To assess impact of mild, moderate and severe hypoglycaemia  and fear of future episodes on patients with T1DM or insulin-treated T2DM | Adults with insulin treated T2DM (n=335)  T1DM (n=202)  insulin T2DM (n=133) | - More T1DM patients reported increased fear of future hypoglycaemia (37.8%) than insulin treated T2DM patients (29.9%). - Subsequent to a severe hypoglycaemic episode, 84.2% of T2DM vs 63.6% of T1DM patients reported greater fear of future hypoglycaemia. - The most common management strategy for hypoglycaemia of any severity was self-treatment. |
|  | Hypoglycaemia | Mitchell [62] | To characterize hypoglycaemic events in T2DM and assess the relationship between the experiences and health outcomes. | T2DM adults (n=1,329) of which:  Insulin T2DM (n=301) | - The prevalence of ≥1 hypoglycaemic event within a 4-week period was 27.5% for the sample overall, higher among insulin users than those not using insulin (43.5% vs. 22.8%, p< 0.0001). - Baseline comparisons showed that worse HbA1c, greater diabetes-related healthcare resource use, greater fear of hypoglycaemia, and impaired health outcomes were associated with experience of hypoglycaemia in the 4weeks prior to baseline. - Hypoglycaemia was associated with worse self-reported glycaemic control, behaviours that contributed to worse glycaemic control, and impairment in patient-reported outcomes. |
|  | Hypoglycaemia  Glycaemic control | Shiu [68] | To examine the relationship between a sense of coherence (SOC), fear of hypoglycaemia and metabolic control to identify whether other variables including  age, hypoglycaemic experience and adherence to self-care practice, confounded the findings from two Swedish studies. | Insulin treated T2DM adults (n=72) | - The higher the SOC (a construct explaining good health and positive adjustment which is on a continuum between “ease” and “disease”), the lower the fear of hypoglycaemia. - There was no significant correlation between SOC and HbA1c. - Findings suggested that respondents with high or low SOC demonstrated no significant difference in difficulties in managing their illness. - The results agreed with that of the Swedish studies that SOC contributes to lower fear of hypoglycaemia. |
|  | Injecting in public | Mehmet [61] | To determine if patients report problems with injecting insulin/SMBG in front of others and explore reasons why. | Insulin T2DM (n=27)  T1DM (n=49) | - Patients reporting problems injecting had T1DM (n=29) T2DM (n=20). - Over 1/3 almost never felt comfortable performing injections/SMBG in public. - 50% almost never inject insulin in front of work colleagues. Most felt comfortable with injections & SMBG in front of family. - Patients of all ages, genders, diabetes type and duration, reported problems injecting & SMBG in front of others. The most common reason was being worried about upsetting or offending others. |
|  | Injection problems | Mollema [63] | To examine functioning and self-management of insulin treated patients suffering from extreme fear of self-injecting (FSI) and/or fear of self-testing (FST). | Patients with insulin treated diabetes (n=1,275) of which:  T1DM (n=740)  T2DM (n=535) | - People with extreme FSI/FST scores compared to the other patients reported higher levels of anxiety and depression. This group also reported more fear of hypoglycaemia and diabetes related distress, had lower levels if general well-being, and reported less frequent SMBG. - A second survey showed 11.1% with extreme FSI/FST scores indicating major depression. - Extreme levels of FSI and/or FST were associated with high diabetes-related distress, poor general well-being, and psychological comorbidity, and poorer treatment adherence to the diabetes treatment. |
|  | Injection problems | Zambanini  [71] | To assess: prevalence of phobia and anxiety-related to insulin injections; association between insulin injection anxiety symptoms with level of general anxiety in the study group; and evaluate their influence of, on glycaemic control. | Insulin treated patients (n=115) of which:  T1DM (n=80) and Insulin T2DM (n=35) | - Patients with T1DM were most likely to inject 4 times a day than those with T2DM (44; 55% vs. 4; 11%) - Injections had been avoided secondary to anxiety in 14% in both T1DM and T2DM. 42% expressed concern at having to inject more frequently. - An injection anxiety score (IAS) of ≥3 was seen in 28% of patients (21 had T1DM [27% of all T1DM] and 12 with T2DM [34% of all T2DM]). Of these, 66% injected insulin 1-2 times a day, and 45% had avoided injections, and 70% would be bothered by more frequent injections. - A general anxiety score (GAS) of ≥8 was seen in 25% (22 had T1DM [27% of all T1DM] and 7 had T2DM [20% of all T2DM]) - No significant correlation was seen with HbA1c and IAS or GAS in T1DM or T2DM. |
|  | Insulin intensification | Cefalu [55] | To understand patients’ perspectives to achieving good glycaemic control and determine how their perceptions of insulin may affect their decisions to initiate or intensify insulin. | T2DM adults (n=1,444)  of which:  Insulin T2DM (n=469) | - 54%-60% were not aware of their recent HbA1c or declined to answer. - Of those on insulin, 120 /175 (69%) reporting HbA1c ≥7%, was unrelated to using pens or syringes. - The majority wished there was another way to take insulin whether currently using insulin or not. - Improving patients’ perceptions and acceptance of insulin could encourage earlier insulin use and assist in achieving and maintaining long term glucose control. |
|  | Insulin adherence | Ary [50] | To assess levels of regime adherence and reasons for non-adherence. | Patients with  T1DM (n=24)  Non-insulin T2DM (n=125)  Insulin T2DM (n=59) | - The top four reasons for insulin non-compliance were being in a bus/plane/car (23%), away from home (13%), being in a restaurant (10%), and on a trip (10%) - Most frequent barriers were being in a bus/plane/car in transit (14%), negative physical reactions (11%) and being in a restaurant (8%) |
|  | Insulin adherence | Peyrot [65] | To examine factors associated with insulin injection omission/ non-adherence | Insulin treated DM adults (n=1,530) of which:  T1DM (n=110)  T2DM (n=1,420) | - 35% reported one or more days of insulin omission/non-adherence. - Omission/non-adherence differed widely across countries (range=20–44%); Most risk factors had similar relationships with insulin omission/non-adherence across countries. - Omission/non-adherence was more frequent among respondents who were male, younger, had T2DM or more frequent hypoglycaemia, were less successful with other treatment tasks, regarded insulin adherence as less important, had more practical/logistical barriers and difficulties with insulin adherence, were concerned that insulin treatment required lifestyle changes or were dissatisfied with flexibility of injection timing. |
|  | Perceptions of T2DM | Mosnier-Pudar [64] | To describe T2DM from the patient’s standpoint in a representative French panel | T2DM Patients (n=1,092)  of which:  Non-Insulin (n=885)  Insulin T2DM (n=207) | - Mean time from diagnosis to insulin was 13.8 years - Disease knowledge improved with treatment intensification and experience. It was greater in insulin-treated patients than in patients with OHAs. - 50% perceived T2DM as serious including those treated with insulin/ - The impact on daily life tended to be greater in patients with longer disease duration, poorer glycaemic control, in women and those treated with insulin. - Patients became partners in their healthcare process and engaged in a more bilateral patient-provider relationship when on insulin   (40% vs. 24%) |
|  |  |  |  |  |  |
| PATIENTS & HCPs | Hypoglycaemia | Brod [51] | To estimate the prevalence of self-treated hypoglycaemia in patients using basal analogues.  To identify demographic treatment-related and behavioural risk factors.  To describe patient and physician responses to these. | T2DM Patients using basal insulin analogues (n=3,042)  Physicians (n=1,222):  Specialists (45%)  PCPs (55%) | - Self-treated hypoglycaemia was common in approximately one third of patients using insulin analogues. - Self-treated hypoglycaemia was associated with clinically significant effects on patient well-being and functioning, patient and physician management and healthcare utilisation. |
|  | Insulin dosing irregularities | Brod [52]  . | To describe basal insulin analogue dosing irregularities; the effect on patient functioning, well-being and management; and the identification of patients most at risk in the study. | T2DM Patients using basal insulin analogues (n=3,042)  Physicians (n=1,222):  Specialists (45%)  PCPs (55%) | - Basal insulin dosing irregularities including missed, mistimed and reduced doses were common. - A significant proportion of patients also report undertaking these irregular dosing behaviours at a frequency that would be considered by prescribers to negatively impact diabetes management. - Physicians reported that the frequency of basal insulin dosing irregularities in the last 30 days that they perceived to have a significant impact on glucose control was missed, mistimed, or reduced   basal insulin doses for patients treated with a basal insulin or a basal-bolus regime. |
|  | Dosing irregularities  Hypoglycaemia | Leiter [60] | To assess the frequency and impact of dosing irregularities and self-treated hypoglycaemia in T2DM patients treated with insulin analogues in the GAPP2 study. | Patients with  Insulin treated T2DM (n=156)  Physicians (n=202)  Of which:  PCPs (n=160)  Specialists (n=42) | - Concern about hypoglycaemia was the most common reason for intentional dosing irregularities by patients. - 26% of patients reported experiencing a dosing irregularity (missed, mistimed or reduced a basal insulin dose) in the previous 30 days. - Up to 60% reported risk for hypoglycaemia as the reason for intentional dosing irregularities. - 80% reported experiencing a self-treated hypoglycaemic event, and 33% recalled having at least one event in the previous month. - HCPs recorded similar levels of patient-reported dosing irregularities. - Over 90% indicated they recommended patients to temporarily reduce their insulin doses to deal with hypoglycaemia. |
|  | Insulin adherence | Peyrot [66] | To examine patient and physician beliefs regarding insulin therapy and degree to which patients adhere to insulin regimes | Insulin treated DM adults (n=1,530)  of which:  T1DM (n=180)  T2DM (n=1,350)  Physicians (n=1,250) of which  Specialists (n=600)  PCPs (n=650) | - More patients reported positive than negative impact on life, except finances (P<0.05) but the trend was stronger for patients with T1DM than with T2DM. - 33.2% of patients reported insulin omission ⁄ non-adherence at least 1 day in the last month with average of 3.3 days. - 72.5% of physicians reported their typical patient does not take their insulin as prescribed, with a mean 4.3 days per month of basal insulin omission ⁄ non-adherence and 5.7 days /month of prandial Insulin omission ⁄ non-adherence. - Patients & providers indicated same five most common reasons for omission⁄ non-adherence: too busy; travelling; skipped meals; stress ⁄ emotional problems; public embarrassment. - Most physicians reported many insulin-treated patients did not have adequate glucose control (87.6%) and would treat more aggressively if not for concern about hypoglycaemia (75.5%). - Though a majority of patients (and physicians) regarded insulin treatment as restrictive, more patients saw insulin treatment as having positive than negative impacts. |
|  | Injection problems | Rubin [67] | To compare patients’ perceptions of injection-related problems with clinicians’ estimates of those problems. | Insulin treated adults (n=501) of which  T2DM (n=385)  PCPs (n=101)  Endocrinologists (n=100)  Diabetes Educators (n=100) | - The majority of patients would like to reduce numbers of injections taken each day. - Almost 50% would be more likely to take insulin regularly if a product were available to ease pain. A smaller proportion reported: injections were serious burden, they were dissatisfied with way of taking insulin, injections had a substantial negative impact on QOL, they skipped injections they should take, or injection-related problems affected injection number they were willing to take. - Awareness of products among HCPs was high, but not effectively communicated to patients. |
|  |  |  |  |  |  |
| HCPs  ONLY | Insulin intensification | Cuddihy [56] | To investigate the opinions of PCPs (primary care professionals) and diabetes specialists on their perceived role in tackling T2DM and the challenges they face, particularly to insulin intensification. | Diabetes specialist physicians (n=300)  PCPs (n=300) | Insulin intensification is defined as “adding a different insulin or an additional injection to the current insulin regimen”   - 21% of all PCPs never initiate and/or modify insulin for T2DM. - Main barriers of insulin intensification cited were lack of experience and lack of time to educate patients. - There was also discord between PCPs and specialists regarding who they considered primarily responsible for insulin intensification. - Better collaboration between primary and secondary care was considered one of the most important factors in improving insulin treatment of T2DM.. |
| HCPs  ONLY | HCP perception of nurse involvement | Siminerio [69] | To examine nurse and  physician perceptions of nurse involvement in diabetes care. | General Nurses(n=51)  DSNs (n=50)  Generalist Physicians (n=166)  Diabetes Specialist Physicians (n=50) | - Nurses & physicians agreed nurses should take a larger role in managing diabetes. - Most common difference identified between nurses & physicians were that nurses provide better education, spend more time with patients, were better listeners, and knew their patients better than physicians. - Specialist nurses talk to patients about self-management, teach medicine management (including insulin therapy), have a higher level of involvement in prescribing, and are more willing to take on additional responsibilities than generalist nurses. |
|  | Insulin management in general practice | Van Avendonk [70] | To investigate the organisation of insulin therapy in general practice and assess factors associated with providing insulin in T2DM patients. | Dutch GPs (n=1,621) | - 67% of GPs start insulin in patients with T2DM. - Male GPs, GPs > 40 years, and GPs working in a health centre are more inclined to start insulin themselves. - GPs working in urban regions less often start insulin than GPs in rural areas. - The most often mentioned barriers for starting and/or monitoring insulin therapy in general practice are lack of knowledge of insulin therapy, lack of time and insufficient financial incentives. - The presence of a practice nurse and diabetes clinics is positively associated with providing insulin therapy in general practice |

Key: DSN = diabetes specialist nurse; PN = practice nurse; GP = general practitioner; HCP = health care professional; NSNHEs = Non-severe nocturnal hypoglycaemic events; OHAs = oral hypoglycaemic agents; PCPs = primary care physicians; QOL = quality of life; SMBG = self-monitoring of blood glucose; T1DM = type 1 diabetes mellitus; T2DM = type 2 diabetes mellitus; Insulin T2DM = insulin treated type 2 diabetes mellitus.
